# Refractive outcomes after cataract surgery in eyes with pterygium: validation of a regression-based keratometric prediction model

**DOI:** 10.3389/fopht.2026.1759853

**Published:** 2026-03-26

**Authors:** Keiji Sato, Ayaka Kawamatsu, Shinya Takahashi, Eri Ishikawa, Yasuhito Ikeda, Toru Kawanobe, Shingo Noda, Yuichiro Tanaka, Tadahiko Kozawa

**Affiliations:** 1 Department of Ophthalmology, Kozawa Eye Hospital and Diabetes Center, Mito, Ibaraki, Japan; 2 Department of Ophthalmology, St. Marianna University School of Medicine, Kawasaki, Kanagawa, Japan

**Keywords:** cataract surgery, corneal radius of curvature, horizontal pterygium size, intraocular lens, refractive error

## Abstract

**Background:**

This study aimed to validate a regression-based keratometric prediction model in eyes with pterygium using actual postoperative refractive outcomes after cataract surgery.

**Methods:**

A retrospective paired analysis was performed in 20 unilateral eyes that underwent staged cataract surgery following pterygium excision. Predicted K values were calculated using a previously reported regression model based on CASIA2 anterior segment parameters. Intraocular lens (IOL) power was calculated using the Barrett Universal II formula. Mean K, spherical equivalent, refractive error, and absolute refractive error were compared among four keratometric methods (postoperative K, predicted K, preoperative K, and fellow-eye K) using the Friedman test followed by Bonferroni-adjusted Wilcoxon signed-rank tests. Sensitivity analyses were conducted after exclusion of toric IOL cases. Exploratory receiver operating characteristic analyses were performed to assess the influence of preoperative corneal parameters.

**Results:**

Overall differences among the four methods were significant for mean K (p < 0.0001), spherical equivalent (p = 0.0003), refractive error (p = 0.001), and absolute refractive error (p = 0.014). However, no statistically significant differences were observed between postoperative K and predicted K in any parameter. Sensitivity analyses yielded consistent findings. Exploratory analyses suggested that preoperative corneal characteristics may influence refractive predictability.

**Conclusion:**

In this paired analysis of 20 unilateral eyes, regression-based K estimation demonstrated refractive performance comparable to postoperative keratometry. These findings support the potential clinical utility of prediction-based keratometry as a practical alternative when postoperative K values are unavailable, although confirmation in larger prospective studies is required.

## Introduction

Pterygium is a fibrovascular overgrowth of conjunctival tissue that often extends onto the nasal cornea and is associated with chronic ultraviolet exposure, dust, and other environmental irritants (1, 2). Its prevalence has also been reported to be higher in hyperopic eyes (3). By altering corneal curvature and inducing corneal astigmatism (4), pterygium may lead to visual impairment (5). Therefore, accurate assessment of corneal shape is essential for intraocular lens (IOL) power calculation in affected eyes. When pterygium excision and cataract surgery are performed simultaneously, postoperative refractive error frequently occurs, often resulting in unintended myopic outcomes (6). Consequently, a staged approach with a minimum interval of three months is generally recommended to allow corneal stabilization (7, 8). Nonetheless, simultaneous surgery may remain necessary in selected patients for whom staged procedures are impractical. Previous studies have evaluated corneal shape changes after pterygium removal and examined methods for estimating postoperative keratometry for IOL calculation (9). However, these investigations have primarily focused on keratometric changes rather than actual postoperative refractive outcomes, which represent the clinically relevant endpoint when assessing IOL calculation accuracy. To our knowledge, direct validation of keratometric prediction formulas using postoperative refractive error as the primary outcome remains limited.

Furthermore, although preoperative keratometry and fellow-eye keratometry are sometimes used as surrogate values when postoperative data are unavailable, their refractive performance has not been systematically compared within the same cohort using a paired analytical framework. We previously proposed a multiple regression model to estimate postoperative keratometry based on CASIA2 anterior segment parameters (10). The present study aimed to evaluate the refractive performance of this prediction model in unilateral eyes undergoing staged cataract surgery after pterygium excision and to directly compare it with postoperative, preoperative, and fellow-eye keratometry using a paired design.

## Materials and methods

### Patients

This retrospective study initially identified 32 patients who underwent pterygium excision followed by staged cataract surgery between November 2020 and September 2021. Twelve patients with bilateral pterygium were excluded to allow paired comparison of all four keratometric methods within the same eye. Consequently, 20 unilateral eyes were included in the primary analysis. The mean age of the patients was 75.3 ± 7.3 years. Four keratometric methods were evaluated within each eye: (i) postoperative K (measured after pterygium excision), (ii) predicted K calculated using the previously reported regression formula, (iii) preoperative K (measured before pterygium excision), and (iv) fellow-eye K (measured from the contralateral eye). Intraocular lens (IOL) power was calculated using the Barrett Universal II (BU II) formula. Mean K and anterior chamber depth (ACD) were measured using the CASIA2 anterior segment optical coherence tomography (OCT) system (Tomey Corporation, Nagoya, Japan). Cataract surgery was performed approximately three months after pterygium surgery. Postoperative subjective spherical equivalent was assessed one month after cataract surgery. Two of the 20 eyes received toric IOLs based on preoperative corneal astigmatism. To evaluate the potential influence of toric implantation, sensitivity analyses were conducted after excluding these cases. Cases with incomplete follow-up (less than three months after pterygium surgery or less than one month after cataract surgery) and cases with recurrent pterygium were excluded.

### Surgical technique

Pterygium surgery was performed under topical anesthesia. The pterygium head was bluntly dissected from the corneal surface at the level of Bowman’s layer, followed by excision of the fibrovascular tissue together with abnormal Tenon’s capsule. A pediculated conjunctival graft was harvested from the inferior bulbar conjunctiva, rotated into position without tension, and secured using interrupted 8–0 Vicryl sutures. Hemostasis was confirmed, and the ocular surface was irrigated with balanced salt solution. Cataract surgery was subsequently performed by an experienced surgeon (E.I.) under topical anesthesia using the Centurion Vision System (Alcon, Fort Worth, TX, USA). A 2.4-mm clear corneal incision was created at the 11-o’clock position, followed by a 7.0-mm continuous curvilinear capsulorhexis. Phacoemulsification was performed using the divide-and-conquer technique, and cortical aspiration was completed thoroughly. Eighteen eyes received monofocal IOLs implanted in the capsular bag using a standard injector system, and two eyes with significant preoperative corneal astigmatism received toric IOLs. The incision was hydrated for closure. No intraoperative complications, including posterior capsule rupture, or postoperative complications, such as endophthalmitis, were observed.

### Clinical data analysis

**Figure 1:**
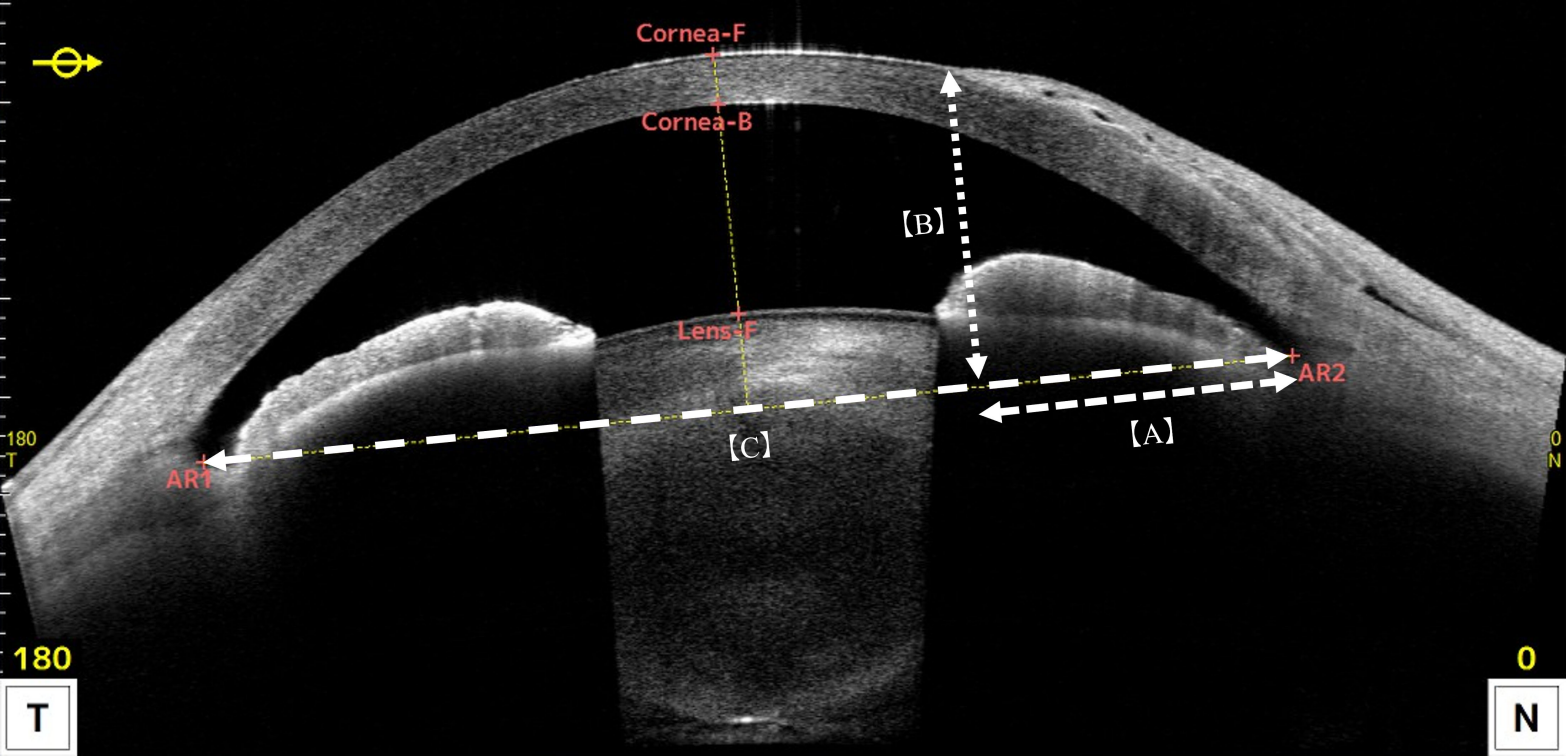
Measurement of horizontal pterygium size (HPS) using CASIA2. A horizontal corneal cross-section obtained with the CASIA2 anterior segment OCT system is shown. The pterygium distance **(A)** was defined as the perpendicular distance from the nasal angle recess to a vertical line **(B)** drawn from the apex of the pterygium. HPS was calculated by dividing this distance by the horizontal angle-to-angle distance **(C)**.


Predicted K values were calculated using the multiple regression equation developed by Takahashi et al. (10). The model incorporates anterior corneal K values measured at the central (A-Center), superior (A-Upper), and inferior (A-Lower) regions, as well as the posterior mean K (P-Ave K) and horizontal pterygium size (HPS), all obtained using the CASIA2 anterior segment OCT system (**Figure 1**). In the horizontal corneal section obtained by anterior segment OCT, the pterygium distance (A) was defined as the perpendicular distance from the nasal angle recess (AR) to a vertical line (B) drawn from the apex of the pterygium to the AR. HPS was calculated as the ratio of the pterygium distance (A) to the horizontal angle-to-angle distance (C). The regression equation used to estimate predicted K was defined as follows:

$$\begin{array}{c} Predicted\;\;K\;=\;0.278\;+\;0.272\;\times (\;A-Center\;K)\;+\\ \;0.276\;\times (A-Upper\;K)\;+\;0.329\;\times (A-Lower\;K)\;+\\ \;0.113\;\times (P-Ave\;K)\;-\;0.410\;\times \;HPS \end{array}$$

### Statistical analyses

All statistical analyses were performed using JMP version 18 (SAS Institute Inc., Cary, NC, USA). The postoperative subjective spherical equivalent measured one month after cataract surgery was defined as the actual postoperative refractive outcome. For each eye, refractive error and absolute refractive error were calculated as the difference between the actual postoperative spherical equivalent and the predicted spherical equivalent obtained by substituting the corresponding K values (postoperative K, predicted K, preoperative K, and fellow-eye K) into both K1 and K2 of the Barrett Universal II formula, while keeping other biometric parameters constant. Because all four keratometric methods were derived from the same eyes, comparisons were conducted using a paired analytical approach. Overall differences among the four methods were assessed using the Friedman test. When significant, *post hoc* pairwise comparisons were performed using Wilcoxon signed-rank tests with Bonferroni correction. Correlations between absolute refractive error and preoperative parameters (horizontal pterygium size [HPS] and preoperative astigmatism) were evaluated using Spearman rank-order correlation analysis. The coefficient of determination (R²) was derived from the correlation coefficient to estimate the strength of association. Absolute refractive error was categorized as <0.5 diopters (D), 0.5–<1.0 D, or ≥1.0 D. An absolute refractive error ≥1.0 D was defined as clinically relevant refractive error. Exploratory univariable logistic regression analyses were performed to assess the associations of HPS and preoperative astigmatism with refractive error (≥1.0 D). Receiver operating characteristic (ROC) curves were generated, and the area under the curve (AUC) was calculated. Cutoff values were determined using the Youden index. Given the limited sample size, ROC analyses were considered exploratory. Sensitivity analyses were performed after excluding eyes implanted with toric IOLs. All p values were two-sided, and p < 0.05 was considered statistically significant.

## Results

**Figure 2:**
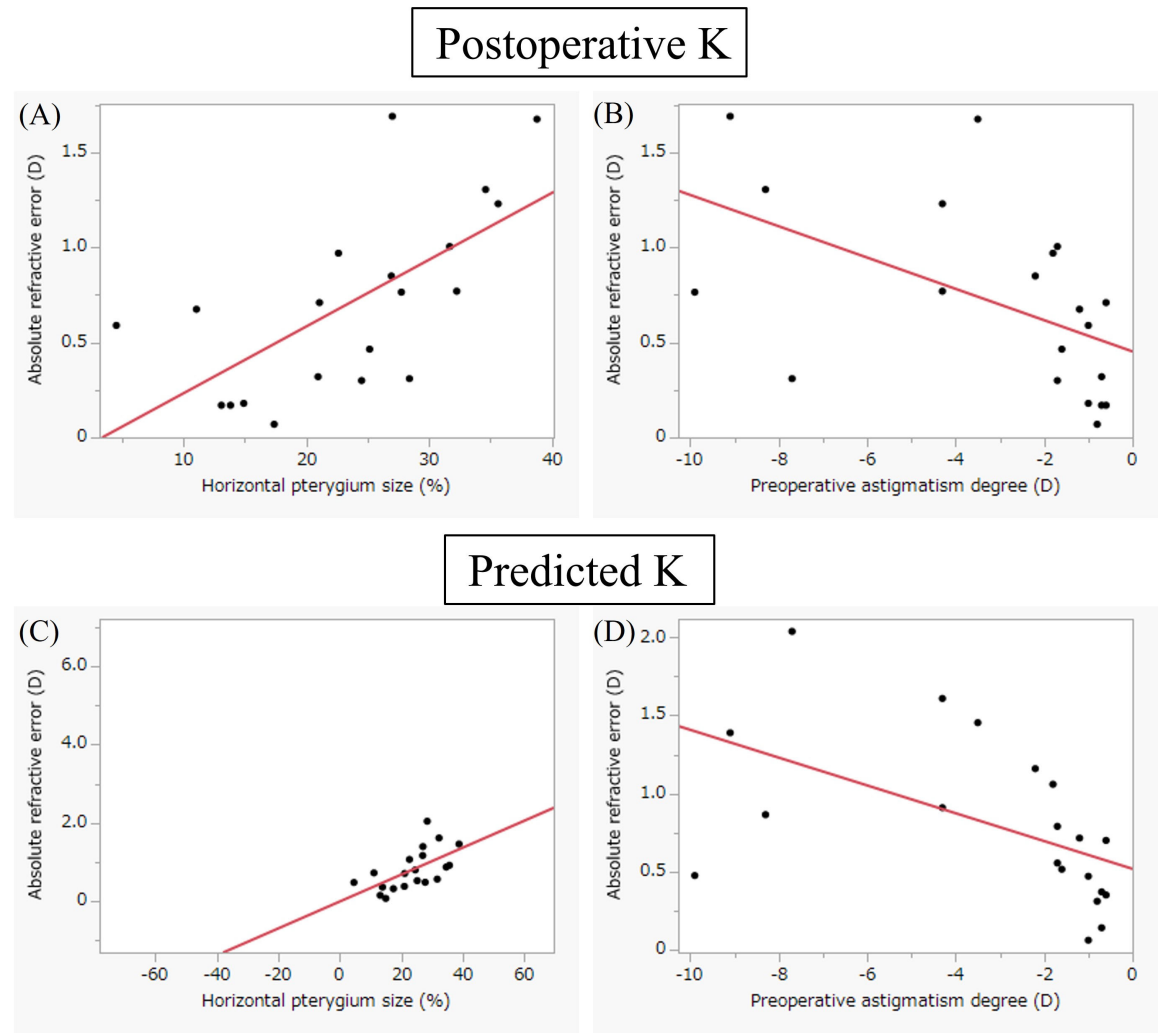
Correlations between absolute refractive error and preoperative parameters. Scatter plots with regression lines illustrate the relationships between absolute refractive error and preoperative horizontal pterygium size (HPS) and preoperative astigmatism for the postoperative K and predicted K methods. In the postoperative K method **(A, B)**, absolute refractive error showed significant correlations with both preoperative HPS (R² = 0.28, p = 0.020) and preoperative astigmatism (R² = 0.42, p = 0.002). In the predicted K method **(C, D)**, significant correlations were also observed with preoperative HPS (R² = 0.28, p = 0.020) and preoperative astigmatism (R² = 0.36, p = 0.005).

**Figure 3:**
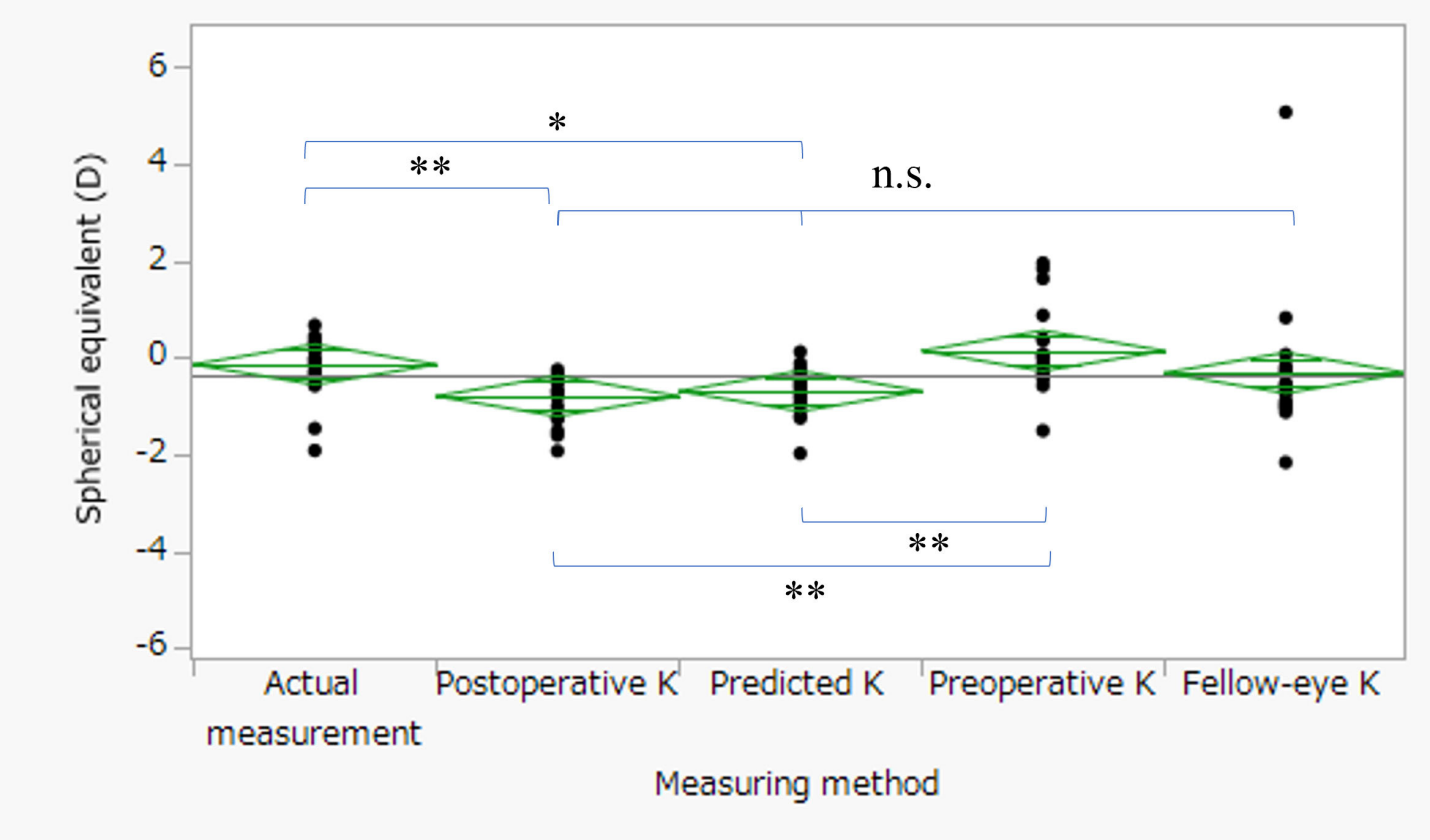
Comparison of measured and predicted spherical equivalent after cataract surgery. Measured postoperative spherical equivalent was compared with predicted values calculated using four keratometric methods: postoperative K, predicted K, preoperative K, and felloweye K. Statistical comparisons were performed using the Friedman test for paired samples, followed by Wilcoxon signed-rank tests with Bonferroni correction for multiple comparisons. The Friedman test demonstrated a significant overall difference among the methods (p < 0.0001). *Post hoc* analysis revealed significant differences between postoperative K and measured values (p < 0.0001), predicted K and measured values (p = 0.01), and between preoperative K and postoperative K (p < 0.0001), as well as between predicted K and preoperative K (p < 0.0001). No statistically significant differences were observed between predicted K and postoperative K (p = 1.00), postoperative K and fellow-eye K (p = 1.00), predicted K and fellow-eye K (p = 1.00), or between preoperative K and measured values (p = 1.00). (Mean K value): *p = 0.01, ** p < 0.0001, n.s. p > 0.05.

**Figure 4:**
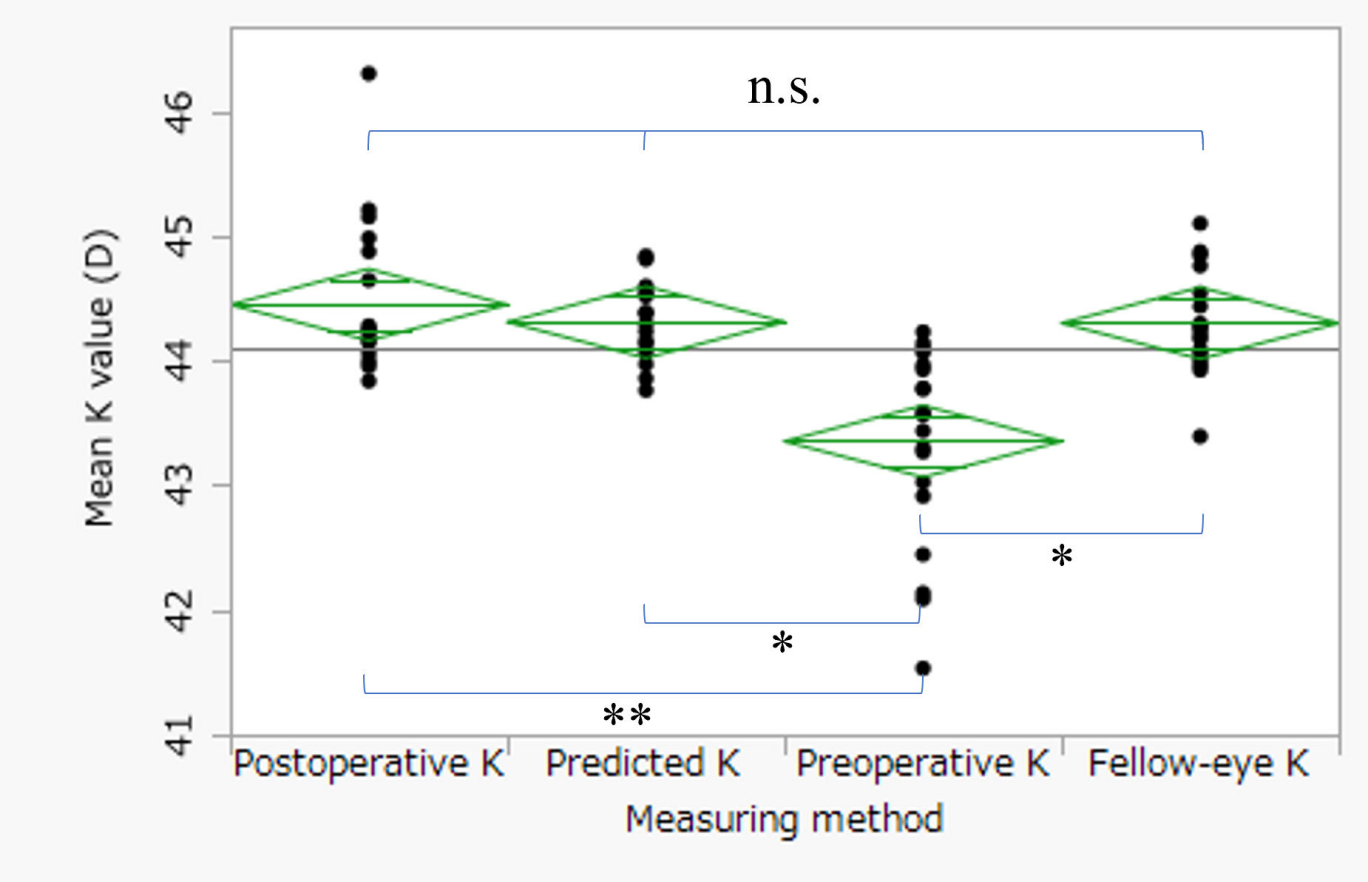
Comparison of mean corneal radius of curvature (K-value) among the four methods. Mean corneal K-values were compared among postoperative K, predicted K, preoperative K, and fellow-eye K using the Friedman test for paired samples, followed by Wilcoxon signedrank tests with Bonferroni correction for multiple comparisons. The Friedman test demonstrated a significant overall difference among the four methods (p < 0.0001). *Post hoc* analysis revealed that preoperative K differed significantly from postoperative K (p < 0.0001), predicted K (p = 0.0006), and fellow-eye K (p = 0.0006). No statistically significant differences were observed among the other pairwise comparisons (all adjusted p = 1.00). *p = 0.0006, ** p < 0.0001, n.s. p > 0.05.

**Figure 5:**
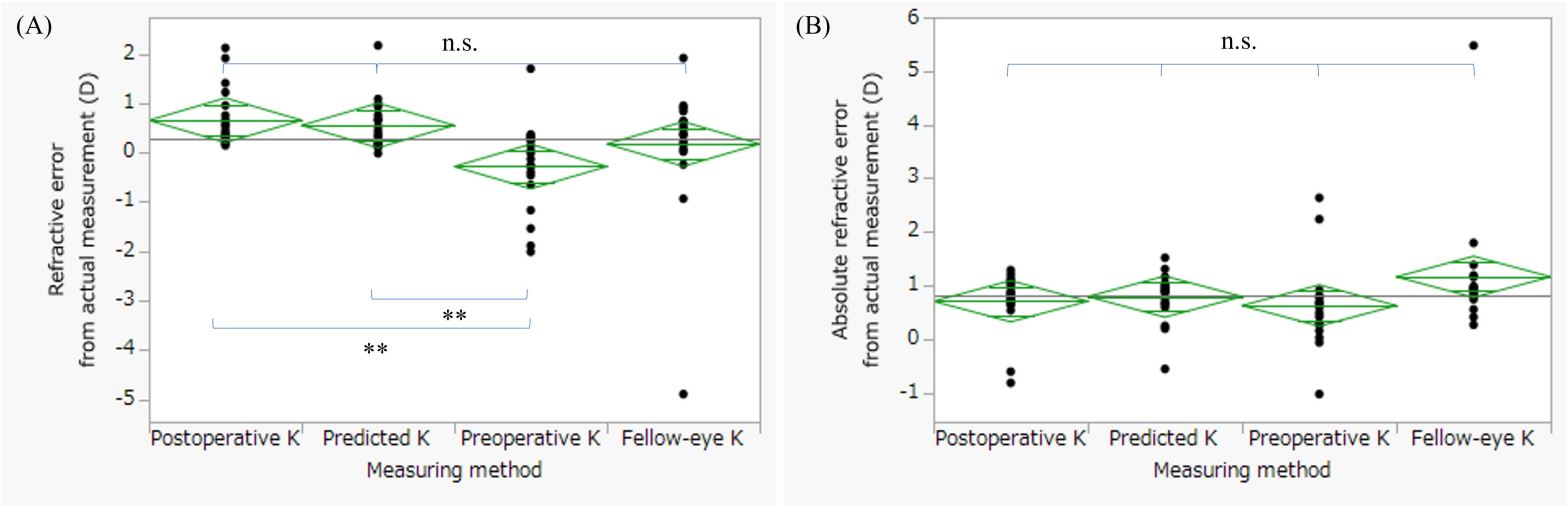
Comparison of refractive error and absolute refractive error among the four keratometric methods. Refractive error **(A)** and absolute refractive error **(B)** were compared among four keratometric methods: postoperative K, predicted K, preoperative K, and fellow-eye K. Statistical analyses were performed using the Friedman test for paired samples, followed by Wilcoxon signed-rank tests with Bonferroni correction for multiple comparisons. For refractive error, the Friedman test demonstrated a significant overall difference among the methods (p = 0.001). *Post hoc* analysis revealed significant differences between preoperative K and postoperative K (p < 0.0001) and between predicted K and preoperative K (p < 0.0001). No statistically significant differences were observed between postoperative K and fellow-eye K (p = 1.00), preoperative K and fellow-eye K (p = 0.193), predicted K and fellow-eye K (p = 1.00), or between predicted K and postoperative K (p = 1.00). For absolute refractive error, the Friedman test also demonstrated a significant overall difference (p = 0.013). However, none of the pairwise comparisons reached statistical significance after Bonferroni correction (postoperative vs fellow-eye, p = 1.00; preoperative vs fellow-eye, p = 0.19; preoperative vs postoperative, p = 0.27; predicted vs fellow-eye, p = 1.00; predicted vs postoperative, p = 1.00; predicted vs preoperative, p = 0.56). ** p < 0.0001, n.s. p > 0.05.

**Figure 6:**
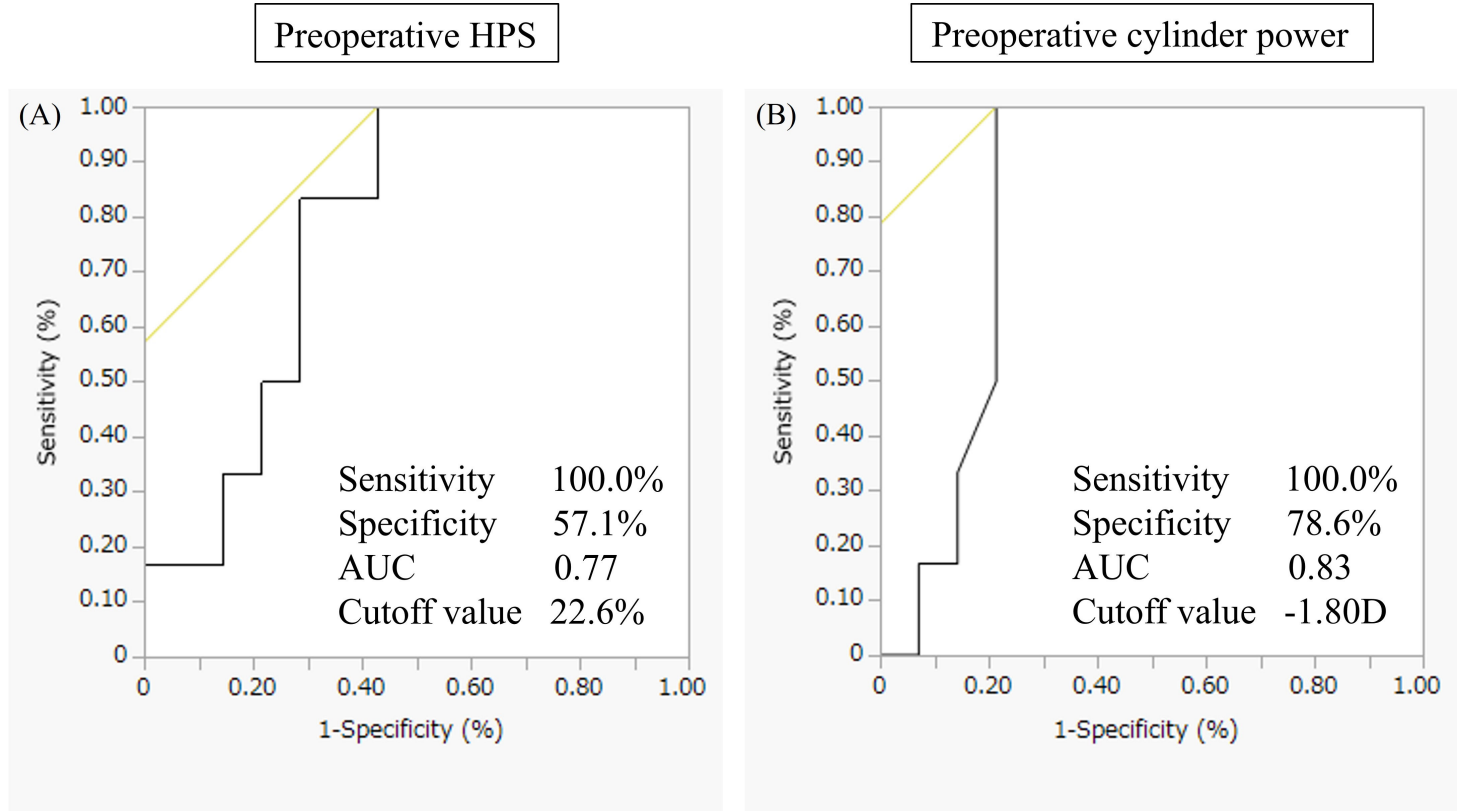
Exploratory ROC curves for preoperative HPS and preoperative astigmatism. Exploratory receiver operating characteristic (ROC) curves derived from univariable logistic regression analyses are shown for preoperative horizontal pterygium size (HPS) **(A)** and preoperative astigmatism **(B)**. For preoperative HPS, the cutoff value was 22.6%, with an area under the curve (AUC) of 0.77, sensitivity of 100%, and specificity of 57.1%. For preoperative astigmatism, the cutoff value was −1.80 D, with an AUC of 0.83, sensitivity of 100%, and specificity of 78.6%. Given the limited sample size, these ROC-derived thresholds should be interpreted as exploratory and hypothesis-generating.

**Figure 2** presents scatter plots illustrating the correlations between absolute refractive error and preoperative parameters. In the postoperative K method (**Figures 2A, B**), absolute refractive error showed significant correlations with both preoperative HPS (R² = 0.28, p = 0.02) and preoperative astigmatism (R² = 0.42, p = 0.002). Similarly, in the predicted K method (**Figures 2C, D**), significant correlations were observed with preoperative HPS (R² = 0.28, p = 0.02) and preoperative astigmatism (R² = 0.36, p = 0.005). **Figure 3** compares mean corneal K values among the four keratometric methods. The Friedman test demonstrated a significant overall difference (p < 0.0001). *Post hoc* Bonferroni-adjusted Wilcoxon signed-rank tests revealed significant differences between preoperative K and postoperative K (p < 0.0001), between preoperative K and predicted K (p = 0.0006), and between preoperative K and fellow-eye K (p = 0.0006). No significant differences were observed among postoperative K, predicted K, and fellow-eye K (all p = 1.00). The mean K values are summarized in **Table 1**. **Figure 4** compares measured postoperative spherical equivalent with values predicted using each keratometric method. The Friedman test demonstrated a significant overall difference (p < 0.0001). *Post hoc* analyses showed that postoperative K (p < 0.0001) and predicted K (p = 0.01) differed significantly from the actual measurement. Preoperative K also differed significantly from postoperative K (p < 0.0001). No significant differences were observed between fellow-eye K and actual measurement (p = 0.63), between preoperative K and actual measurement (p = 1.00), or among postoperative K, predicted K, and fellow-eye K (all p = 1.00). Detailed values are presented in **Table 2**. **Figure 5** illustrates refractive error and absolute refractive error across the four keratometric methods. Refractive error is shown in **Figure 5A** and absolute refractive error in **Figure 5B**. For refractive error (**Figure 5A**), the Friedman test indicated a significant overall difference (p = 0.001). *Post hoc* comparisons demonstrated significant differences between preoperative K and postoperative K (p < 0.0001) and between preoperative K and predicted K (p < 0.0001). No other pairwise comparisons reached statistical significance. For absolute refractive error (**Figure 5B**), the Friedman test also indicated a significant overall difference (p = 0.014); however, no significant pairwise differences were observed after Bonferroni correction. Summary statistics are provided in **Tables 3** and **4**. The distribution of absolute refractive error categories is shown in **Table 5**. The proportions of eyes achieving an absolute refractive error <1.0 D were 75.0% for postoperative K, 70.0% for predicted K, 85.0% for preoperative K, and 55.0% for fellow-eye K. Exploratory ROC analyses were performed to evaluate the ability of preoperative HPS and preoperative astigmatism to discriminate eyes with refractive error ≥1.0 D (**Figures 6A, B**). For HPS (**Figure 6A**), the AUC was 0.77 with a cutoff value of 22.6% (sensitivity 100%, specificity 57.1%). For preoperative astigmatism (**Figure 6B**), the AUC was 0.83 with a cutoff value of −1.80 D (sensitivity 100%, specificity 78.6%). Given the limited sample size, these findings should be interpreted as exploratory. Sensitivity analyses excluding the two eyes implanted with toric IOLs (n = 18) yielded consistent findings. The Friedman test demonstrated significant overall differences for mean K (p < 0.0001), spherical equivalent (p = 0.0003), refractive error (p = 0.001), and absolute refractive error (p = 0.014). Bonferroni-adjusted Wilcoxon signed-rank tests confirmed no significant differences between postoperative K and predicted K for any parameter (all p = 1.00).

**Table 1:** Mean corneal radius of curvature (K-value) among the four keratometric methods.

Methods	N	Mean K-value (95% CI)
Postoperative K	20	44.5 ± 1.74 (43.6–45.3)
Predicted K	20	44.3 ± 1.66 (43.5–45.1)
Preoperative K	20	43.4 ± 2.01 (42.4–44.3)
Fellow-eye K	20	44.3 ± 1.65 (43.5–45.1)

Values are presented as mean ± standard deviation (SD) with 95% confidence intervals (CI).

**Table 2:** Measured and predicted spherical equivalent among the four keratometric methods.

Methods	N	Spherical equivalent (95% CI)
Actual measurement	20	− 0.13 ± 0.82 (− 0.52–0.25)
Postoperative K	20	− 0.79 ± 0.80 (− 1.17–− 0.42)
Predicted K	20	− 0.69 ± 1.00 (− 1.16–− 0.22)
Preoperative K	20	0.14 ± 1.32 (− 0.47–0.76)
Fellow-eye K	20	− 0.31 ± 1.95 (− 1.22–0.60)

Values are presented as mean ± standard deviation (SD) with 95% confidence intervals (CI).

**Table 3:** Refractive error relative to the actual postoperative measurement among the four keratometric methods.

Methods	N	Refractive error (95% CI)
Postoperative K	20	0.66 ± 0.56 (0.39–0.92)
Predicted K	20	0.55 ± 0.78 (0.18–0.92)
Preoperative K	20	− 0.28 ± 1.10 (− 0.79–0.23)
Fellow-eye K	20	0.18 ± 1.84 (− 0.69–1.00)

Values are presented as mean ± standard deviation (SD) with 95% confidence intervals (CI).

**Table 4:** Absolute refractive error relative to the actual postoperative measurement among the four keratometric methods.

Methods	N	Absolute refractive error (95% CI)
Postoperative K	20	0.71 ± 0.49 (0.48–0.94)
Predicted K	20	0.80 ± 0.52 (0.55–1.04)
Preoperative K	20	0.63 ± 0.93 (0.19–1.07)
Fellow-eye K	20	1.17 ± 1.41 (0.51–1.83)

Values are presented as mean ± standard deviation (SD) with 95% confidence intervals (CI).

**Table 5:** Distribution of absolute refractive error among the four keratometric methods.

Methods	<0.5 D, n (%)	0.5–<1.0 D, n (%)	≥1.0 D, n (%)
Postoperative K	8 (40.0)	7 (35.0)	5 (25.0)
Predicted K	7 (35.0)	7 (35.0)	6 (30.0)
Preoperative K	13 (65.0)	4 (20.0)	3 (15.0)
Fellow-eye K	7 (35.0)	4 (20.0)	9 (45.0)

Values represent the number of eyes (n) and corresponding percentages (%).

## Discussion

Although previous studies have reported the usefulness of predicted K values after pterygium excision for IOL power calculation in simultaneous pterygium and cataract surgery, their clinical accuracy has remained uncertain because actual postoperative refractive outcomes were not evaluated. The present study addressed this gap by validating a regression-based keratometric prediction formula using refractive outcomes from staged cataract surgery following pterygium excision. To our knowledge, this represents one of the first refractive-outcome–based validations of a keratometric prediction model in eyes with pterygium, thereby providing clinically meaningful evidence beyond keratometric change alone. **Figure 2** demonstrated that absolute refractive error was significantly correlated with both preoperative HPS and preoperative astigmatism in the postoperative K and predicted K methods. These findings suggest that baseline corneal morphology and astigmatic magnitude influence refractive predictability regardless of the keratometric source used for IOL calculation. The persistence of these correlations even when postoperative K values were used indicates that residual refractive variability may reflect intrinsic corneal structural characteristics rather than solely inaccuracies in keratometric estimation. In the predicted K method, incorporation of HPS into the regression model (10) further supports the biological plausibility of these associations. **Figure 3** demonstrated a significant overall difference in mean corneal K values among the four keratometric methods. Preoperative K differed significantly from postoperative K, predicted K, and fellow-eye K, whereas no significant differences were observed between postoperative K and predicted K. These findings indicate that preoperative keratometry systematically underestimates corneal power in eyes affected by pterygium. Importantly, the absence of a significant difference between postoperative K and predicted K suggests that regression-based K estimation can approximate postoperative keratometry in a clinically acceptable manner.

Postoperative refractive error in eyes with corneal irregularity is increasingly recognized to arise from inaccurate estimation of effective corneal power rather than intrinsic limitations of modern IOL calculation formulas. Conventional keratometry assumes a fixed anterior–posterior curvature ratio and regular corneal geometry, assumptions that may not hold in eyes with pterygium-induced structural distortion. In such cases, optimizing corneal power estimation may be more impactful than modifying the IOL formula itself (11). Recent investigations have emphasized integration of anterior and posterior corneal measurements and the development of multivariate or machine-learning–based prediction strategies to improve refractive accuracy in complex corneas (12, 13). Within this framework, the Takahashi regression formula represents a clinically practical approach to enhanced corneal power estimation while maintaining feasibility in routine practice. In **Figure 4**, both postoperative K and predicted K differed significantly from the actual postoperative spherical equivalent. These findings suggest that small systematic refractive shifts may persist when these K values are applied within the Barrett Universal II formula. The observed refractive tendency is consistent with previous reports indicating that the Barrett Universal II formula may exhibit a mild hyperopic shift in eyes with shallow anterior chamber depth or specific biometric profiles (14). Notably, no significant difference was observed between postoperative K and predicted K, reinforcing that regression-based K estimation performs comparably to postoperative keratometry in terms of spherical equivalent prediction. In contrast, the greater variability observed in the fellow-eye K method suggests reduced reliability in eyes with asymmetric corneal remodeling. **Figure 5** demonstrated that although refractive error differed significantly among methods, particularly between preoperative K and both postoperative K and predicted K, absolute refractive error did not differ significantly after correction for multiple comparisons. This indicates that while the direction of refractive bias may vary, the magnitude of error is broadly comparable between postoperative K and predicted K. Although the proportion of eyes achieving absolute refractive error <1.0 D was numerically highest in the preoperative K method, this did not translate into statistically superior accuracy and likely reflects sample size limitations and variability. The fellow-eye K method showed both lower success rates and greater dispersion, underscoring the limitations of surrogate keratometric approaches in eyes with asymmetric corneal changes. Given that pterygium size strongly influences corneal astigmatism (15), both HPS and preoperative astigmatism appear to be important determinants of refractive predictability. Exploratory ROC analyses yielded moderate discriminatory ability (AUC 0.77–0.83). These findings are consistent with prior studies demonstrating increased refractive surprise when pterygium extension exceeds certain thresholds (16, 17) and highlighting the impact of pterygium-induced astigmatism on IOL calculation (18). However, given the limited sample size and perfect sensitivity observed in this cohort, these cutoff values should be regarded as hypothesis-generating. Sensitivity analyses excluding toric IOL cases yielded consistent findings, further supporting the robustness of the primary conclusions. Importantly, additional analyses limited to non-toric eyes demonstrated results identical to the main analysis, confirming that toric implantation did not materially confound the comparison between postoperative and predicted keratometry. Several limitations warrant consideration. First, this retrospective study included a relatively small sample of unilateral eyes, limiting statistical power and generalizability. Second, postoperative refraction was assessed one month after cataract surgery; longer follow-up may be required to confirm refractive stability. Third, although toric IOL implantation was accounted for in sensitivity analyses, residual confounding cannot be entirely excluded. Finally, ROC analyses were exploratory and require validation in larger prospective cohorts. In conclusion, regression-based K-value prediction demonstrated refractive performance comparable to postoperative keratometry in eyes undergoing staged cataract surgery after pterygium excision. While not superior, prediction-based keratometry provided clinically acceptable accuracy and may serve as a practical alternative when postoperative keratometry is unavailable.

### Limitation

This study has several limitations. First, its retrospective design and relatively small sample size (20 unilateral eyes) limit statistical power and generalizability. In particular, the exploratory ROC analyses may be susceptible to overfitting and should therefore be interpreted with caution. Second, postoperative refraction was assessed one month after cataract surgery; longer follow-up would be necessary to confirm long-term refractive stability in eyes previously affected by corneal remodeling. Third, although only two eyes received toric IOL implantation and sensitivity analyses yielded consistent results, the possibility of residual confounding cannot be entirely excluded. Finally, the study was conducted at a single center using a specific imaging device and IOL calculation formula, which may limit the generalizability of these findings to other clinical environments or alternative formula platforms.

## Conclusion

In this paired analysis of 20 unilateral eyes, regression-based predicted K demonstrated refractive performance comparable to postoperative K, with no significant difference in absolute refractive error between the two methods. Although overall differences were observed among keratometric approaches, *post hoc* analyses confirmed that predicted K approximated postoperative keratometry in a clinically meaningful manner. Exploratory findings suggest that preoperative corneal characteristics, including horizontal pterygium size and preoperative astigmatism, may influence refractive predictability; however, these observations require validation in larger prospective cohorts. Within these limitations, regression-based keratometric estimation may serve as a practical alternative when postoperative keratometry is unavailable.

## Data Availability

The datasets presented in this study can be found in online repositories. The names of the repository/repositories and accession number(s) can be found in the article/**Supplementary Material**.

## Supplementary material

Supplementary Table 1 Raw data of the study participants.
