# Supplementary material for: Refractive outcomes after cataract surgery in eyes with pterygium: validation of a regression-based keratometric prediction model
Source: Front Ophthalmol (Lausanne). 2026 Mar 26;6:1759853. doi: 10.3389/fopht.2026.1759853 (PMC13061672; doi:10.3389/fopht.2026.1759853)
Supplement: Supplementary Table 1 — Raw data of the study participants. [file Table1.docx]

**Supplementary Material**

**AI Prompts Provided to the Generative AI Tool**

**First Prompt Used**

“I would like to improve the introduction and discussion sections of my manuscript. Please provide critical feedback from the perspective of a top-tier peer reviewer.”

**Last Prompt Used**

“Please create a finalized disclosure statement regarding the use of generative AI, following the requirements of Frontiers.”

**Notes**

No additional prompts were used beyond those listed above.
The generative AI tool (ChatGPT, OpenAI GPT-5.1, <https://chat.openai.com/>) assisted only with language refinement and editorial clarity.
All scientific content, data analysis, interpretation, and conclusions were produced entirely by the authors.
